# In silico identification of HDAC3 inhibitors for potential Alzheimer’s disease therapeutics

**DOI:** 10.1002/alz70859_104774

**Published:** 2025-12-25

**Authors:** Thabo Brighton Makgoba, Jacques Joubert, Erika Kapp

**Affiliations:** ^1^ University of the Western Cape, Cape Town, Western Cape South Africa; ^2^ University of the Western Cape, Cape Town South Africa

## Abstract

**Background:**

Histone deacetylases (HDACs) are epigenetic enzymes linked to several biological functions and diseases. Among HDACs, the class I enzymes show high similarity in protein sequence and active site. Non‐selective inhibition of HDACs has side effects. However, HDAC3‐selective inhibitors in preclinical studies showed promise in non‐communicable diseases with fewer complications. Most HDAC3‐selective inhibitors contain a benzamide zinc‐binding group (ZBG). Hydroxamates are highly potent HDAC inhibitors, but they exhibit toxicity, poor pharmacokinetic and selectivity profiles. Benzamide‐based inhibitors offer improved selectivity but lack potency and often optimisation compromises potency. There is a need for HDAC3 inhibitors with novel ZBGs, balanced potency and selectivity, while maintaining good safety and pharmacokinetic profiles. This study aims to identify HDAC3‐selective inhibitors with novel ZBGs using in silico techniques.

**Method:**

The in silico tools PROCHECK, VERIFY3D, Molecular Operating Environment, and ProteinsPlus, were utilised to validate HDAC3 protein structure, active site prediction and consensus confirmation, and predict zinc ion coordination geometry. Schrodinger Maestro was used for protein and ligand preparation, and molecular docking. Benchmarking was performed through re‐docking and docking of actives and decoys to evaluate and validate the docking program, docking parameters and algorithm. Structure‐based virtual screening with predefined criteria identified 22 structurally diverse compounds from focused libraries for purchase and subsequent in vitro evaluation.

**Result:**

Out of the 22 compounds tested against HDAC3, 11 compounds showed inhibitory activity. Compound **a** showed the highest inhibitory activity (89.93%), compound **k** showed the lowest inhibitory activity (0.61%) at a 20 µM screening concentration. Compound **a** showed IC50 of 0.99 µM against HDAC3 but it showed poor selectivity against HDAC1 and HDAC2. Compound **b** showed an IC50 of 15.5 µM and it showed 7.4‐ and 5.7‐fold selectivity against HDAC1 and 2 respectively. Neither compound inhibited HDAC8, and they were predicted to be blood brain barrier (BBB) impermeant. Toxicity predictions showed that compound **a** may cause neurotoxicity and respiratory toxicity and compound **b** may cause neurotoxicity and hepatotoxicity.

**Conclusion:**

The study identified novel compounds with HDAC3 inhibition. However, it also highlighted the need for further structural optimisation to improve inhibitory activity, maintain selectivity, ensure BBB permeability and reduce toxicity.

